# The impact of COVID-19 status and vaccine type following the first dose on acute heart disease: A nationwide retrospective cohort study in South Korea

**DOI:** 10.1017/S0950268824001213

**Published:** 2024-10-24

**Authors:** Choa Yun, Yaeji Lee, Seok-Jae Heo, Namhui Kim, Inkyung Jung

**Affiliations:** 1 Department of Biostatistics and Computing, Yonsei University, Seoul, South Korea; 2 Division of Biostatistics, Department of Biomedical Systems Informatics, Yonsei University College of Medicine, Seoul, South Korea

**Keywords:** adverse event, heart disease, SARS-CoV-2 infection, vaccine safety surveillance

## Abstract

Recent studies have suggested an increased incidence of myocarditis and pericarditis following mRNA vaccination or COVID-19. However, the potential interaction effect between vaccine type and COVID-19 on heart disease risk remains uncertain. Our study aimed to examine the impact of COVID-19 status and vaccine type following the first dose on acute heart disease in the Korean population, using data from the National Health Insurance Service COVID-19 database (October 2018–March 2022). We sought to provide insights for public health policies and clinical decisions pertaining to COVID-19 vaccination strategies. We analysed heart disease risk, including acute cardiac injury, acute myocarditis, acute pericarditis, cardiac arrest, and cardiac arrhythmia, in relation to vaccine type and COVID-19 within 21 days after the first vaccination date, employing Cox proportional hazards models with time-varying covariates. This study included 3,350,855 participants. The results revealed higher heart disease risk in individuals receiving mRNA vaccines than other types (adjusted HR, 1.48; 95% CI, 1.35–1.62). Individuals infected by SARS-CoV-2 also exhibited significantly higher heart disease risk than those uninfected (adjusted HR, 3.56; 95% CI, 1.15–11.04). We found no significant interaction effect between vaccine type and COVID-19 status on the risk of acute heart disease. Notably, however, younger individuals who received mRNA vaccines had a higher heart disease risk compared to older individuals. These results may suggest the need to consider alternative vaccine options for the younger population. Further research is needed to understand underlying mechanisms and guide vaccination strategies effectively.

## Introduction

As of April 2023, the coronavirus disease 2019 (COVID-19) continues to have a global impact. Unlike previous pandemics, significant progress has been made in biotechnology, leading to the successful development of vaccines for COVID-19 on multiple platforms. Vaccination efforts commenced in the United States in December 2020 and were later initiated in South Korea on 28 January 2021. The initial phase of vaccination in South Korea, starting in February 2021, targeted 60,000 healthcare workers directly involved in the COVID-19 response. Subsequently, vaccinations were extended to the elderly residing in nursing homes and long-term care facilities, as well as inpatients and mental rehabilitation hospital workers. In May, high-risk groups, including individuals aged 65 and older, disabled individuals, and healthcare professionals, began receiving vaccinations. The rest of the population became eligible for vaccination from July onwards. The government of the South Korea set a goal to achieve herd immunity by vaccinating 70% of the population. This goal was realized by completing the first vaccination for at least 70% of the population by the end of October 2021. The vaccines introduced in South Korea include viral vector vaccines (manufactured by AstraZeneca and Johnson & Johnson (JNJ)), mRNA vaccines (manufactured by Pfizer/bioNTech and Moderna), and a protein subunit vaccine (manufactured by NovaVax) [1].

The BNT162b2 vaccine (Pfizer/bioNTech) and mRNA-1273 vaccine (Moderna) take a new approach to vaccination, utilizing mRNA technology as their active agent. Unlike traditional vaccines that rely on live-attenuated, inactivated, or a singular antigen of the virus, new methods are currently under research, including mRNA-based vaccines, viral vectors, DNA-based vaccines, and antigen-presenting cell injections [2]. The BNT162b2 vaccine showed 95% efficacy over an average of 2-month follow-up period, leading to emergency approval by the U.S. Food and Drug Administration (FDA) [3, 4]. Initially approved for individuals aged 16 and older, the vaccine is administered in two doses with a 21-day interval. Similarly, the mRNA-1273 vaccine exhibited an efficacy of 94.1% [5]. However, there have been reports of pericarditis and myocarditis following COVID-19 vaccination, particularly in adolescents and young adult males, occurring within several days after receiving mRNA-based COVID-19 vaccines (Pfizer/bioNTech or Moderna) [6]. These cases are actively monitored by the Centers for Disease Control and Prevention (CDC) and its partners through data review and medical record assessment to understand the relationship to COVID-19 vaccination [7]. Recent studies have shown that the risk of myocarditis and pericarditis seems highest after the second dose of mRNA vaccines [8–12].

In South Korea, myocarditis and pericarditis following COVID-19 vaccination are monitored through active surveillance by the Korea Disease Control and Prevention Agency (KDCA). Healthcare providers report cases to the KDCA, which investigates for any causal relationship with vaccination. Epidemiological studies may also be conducted to assess incidence and risk factors. As research continues, mRNA vaccines represent a promising advancement in the fight against COVID-19, but safety considerations, especially in specific populations, remain crucial.

COVID-19 results from the infection of cells by the severe acute respiratory syndrome coronavirus 2. This virus enters cells by binding to the angiotensin-converting enzyme 2 receptor [13]. There is a high prevalence of cardiovascular disease among patients with COVID-19, with more than 7% of patients experiencing myocardial injury from the infection [14]. Among young male adolescents and adults, there were 11.54 additional events per 100,000 individuals attributed to this increased risk of myocarditis after infection [15]. The primary mechanisms that lead to heart damage in COVID-19 appear to be direct harm to heart muscle cells due to viral infection and the impact of widespread inflammation throughout the body. The most prevalent cardiac abnormality observed in COVID-19 is acute cardiac injury, defined as a notable increase in cardiac troponin levels, and this is seen in around 8–12% of all patients [16].

However, limited research has examined the interaction between COVID-19 and vaccine type on heart disease occurrence. Our study aimed to evaluate the impact of COVID-19 status and vaccine type following the first dose on acute heart disease in the Korean population, using data from the National Health Insurance Service (NHIS) COVID-19 database (October 2018–March 2022). We hypothesized that adverse events (AEs) might differ based on the type of vaccines, COVID-19 status, or a combination of both. Additionally, we investigated how age might interact with the type of COVID-19 vaccines on the occurrence of acute heart disease. Understanding the interaction between vaccine type and COVID-19 status in influencing acute heart disease could assist clinicians in customizing vaccination recommendations for their patients.

## Methods

### Data and study population

The data used in this study were obtained from the NHIS COVID-19 database, covering the period from October 2018 to March 2022. This comprehensive database comprises medical claims of 10,663,738 individuals, representing approximately 24% of the South Korean population who received one or more vaccines from February 2021 to March 2022. The NHIS COVID-19 database is a cohort dataset comprising randomly sampled vaccine recipients, connecting NHIS claim data from October 2018 to March 2022 with individuals who received vaccinations between February 2021 and March 2022. We identified acute heart disease cases using the codes from the 10th revision of the International Statistical Classification of Diseases and Related Health Problems (ICD-10) codes. To focus on newly diagnosed cases after vaccination, individuals diagnosed with acute heart disease before their vaccination date were excluded. The exclusion criteria included: (1) No records of the first dose vaccination date; (2) Outcome events occurring before the first vaccination date; and (3) COVID-19 reported prior to the first vaccination date. Following these exclusions, the total population considered in this study comprised 3,350,855 individuals.

### Ethical consideration

This study did not require informed consent from individual participants as we used a de-identified dataset and received an exemption from the Institutional Review Board of Severance Hospital (IRB: 4-2022-0594).

### Outcomes and Covariates

The primary outcome variable in this study was the occurrence of acute heart diseases. The acute heart diseases included acute cardiac injury (ICD-10: I21), acute myocarditis (ICD-10: I40), acute pericarditis, unspecified (ICD-10: I309), cardiac arrest, unspecified (ICD-10: I469), and cardiac arrhythmia, unspecified (ICD-10: I499). We extracted the date of the initial diagnosis of acute heart diseases and defined occurrences within 21 days after the first vaccination date as potential AEs related to vaccination.

The variables of interest were the type of vaccine (mRNA/Others) and the COVID-19 status (yes/no). The mRNA vaccines consisted of BNT162b2 (Pfizer/bioNTech) and mRNA-1273 (Moderna). Other vaccines included AZD1222 (AstraZeneca), JNJ-78436735 (Janssen), and NVX-CoV2373 (Novavax). The COVID-19 status was defined as ‘yes’ if the individual was infected within 21 days after the first vaccination date, with a positive result in the real-time polymerase chain reaction (PCR) test. Note that the COVID-19 status was considered a time-varying covariate in the regression model, as it could change during the follow-up period.

The other covariates included sex, age, and Charlson’s comorbidity index (CCI). Age at the first vaccination date was grouped into 10-year intervals (10–19, 20–29, 30–39, 40–49, 50–59, 60–69, and 70+ years). CCI was calculated by summing points assigned to each comorbid condition identified from the ICD-10 codes recorded within 1 year before the first dose vaccination date and categorized into 0, 1, and 2+.

### Statistical analysis

Categorical variables were presented as counts and percentages and compared using the χ2 test. We used Cox proportional hazards regression to model the risk of acute heart disease. Time to occurrence of acute heart disease was defined as the interval from the date of the first vaccination to the date of the first acute heart disease diagnosis, as recorded in the medical records. It was censored by the last follow-up date or death. We first examined the effects of vaccine type and COVID-19 status both with and without adjustment for covariates. Subsequently, we incorporated the interaction effect between vaccine type and COVID-19 status into the regression model. Potential confounding covariates, such as sex, age group, and CCI, were also included in the analysis. Furthermore, we examined the interaction effect between the type of vaccine and age group. All statistical analyses were performed using SAS version 7.1 Enterprise (SAS Institute, Cary, NC), and all P-values were two-sided, with a significance level of 0.05.

## Results

The study cohort included 3,350,855 participants. Table 1 shows the descriptive statistics of the general characteristics of the study population by the type of vaccine and COVID-19 status. Among the participants, 62.48% (2,093,700) received the mRNA vaccine, while 37.52% (1,257,155) received other vaccines. Among those who received the mRNA vaccine, 0.07% (1,388) had COVID-19 within 21 days after the first vaccination, and 0.02% (194) among those who received other vaccines. The incidence of acute heart disease, as well as sex, age group, and CCI distributions, significantly differed among the four groups by the vaccine type and COVID-19 status (P < .0001). More specifically, the incidence of acute heart disease was higher among individuals with COVID-19 in both vaccine groups, with a p-value of 0.0090 for the mRNA vaccine group (data not shown). However, this difference did not reach statistical significance among those who had received other vaccines, where the p-value was 0.2460. Among those who received mRNA vaccines, a significantly higher proportion of males were infected (p < 0.0001). Additionally, younger age groups had a higher frequency of COVID-19 compared to older age groups (p < 0.0001). This may account for the observed lower CCI among infected individuals in the mRNA vaccine group (p < 0.0001).

**Table 1.** General characteristics of study population by vaccine type and COVID-19 status^c^

	mRNA vaccine^a^ (n = 2,093,700, 62.48%)		Others^b^ (n = 1,257,155, 37.52%)		
	Infected (n = 1,388, 0.07%)	Not infected (n = 2,092,312, 99.93%)	Infected (n = 194, 0.02%)	Not infected (n = 1,256,961, 99.98%)	
	n (%)	n (%)	n (%)	n (%)	P–value
*Occurrence of heart disease*					<.0001
Yes	6 (0.43)	2,679 (0.13)	1 (0.52)	1,078 (0.09)	
No	1,382 (99.57)	2,089,633 (99.87)	193 (99.48)	1,255,883 (99.91)	
*Sex*					<.0001
Male	605 (43.59)	796,673 (38.08)	76 (39.18)	529,577 (42.13)	
Female	783 (56.41)	1,295,639 (61.92)	118 (60.82)	727,384 (57.87)	
*Age group*					<.0001
10–19	147 (10.59)	126,143(6.03)	0 (0.00)	43 (0.00)	
20–29	254 (18.30)	273,981 (13.09)	1 (0.52)	11,341(0.90)	
30–39	223(16.07)	257,933 (12.33)	24 (12.37)	90,235 (7.18)	
40–49	291 (20.97)	370,719 (17.72)	29 (14.95)	35,161 (7.57)	
50–59	372 (26.80)	541,629 (25.89)	18 (9.28)	103,755 (8.25)	
60–69	34 (2.45)	58,842 (2.81)	95 (48.97)	683,796 (54.40)	
70+	67 (4.83)	463,065(22.13)	27 (13.92)	272,630 (21.69)	
*Charlson comorbidity index*					<.0001
0	1,228 (88.47)	1,728,506 (82.61)	159 (81.96)	972,352 (77.36)	
1	138 (9.94)	305,439 (14.60)	29 (14.95)	236,746 (18.83)	
2+	22 (1.59)	58,367 (2.79)	6 (3.09)	47,863 (3.81)	

a The mRNA vaccines consist of BNT162b2 and mRNA-1273.

b Other vaccines include AZD1222 (AstraZeneca), JNJ-78436735 (Janssen), and NVX-CoV2373 (Novavax).

c COVID-19 status was defined as yes if infected before 21 days after the first dose vaccination date.

We presented the hazard ratios (HRs) for vaccine type, COVID-19 status, and the other covariates in Table 2. Individuals who received mRNA vaccines had a higher risk of acute heart disease compared to those who received other vaccines (unadjusted HR, 1.49; 95% CI, 1.39–1.60; P < .0001, and adjusted HR, 1.48; 95% CI, 1.35–1.62; P < .0001). The risk of acute heart disease was higher among individuals who had COVID-19 within 21 days after the first vaccination than among those whom this was not registered (unadjusted HR, 4.08; 95% CI, 1.31–12.64; P = 0.0150, and adjusted HR, 3.56; 95% CI, 1.15–11.04; P = 0.0280). Younger individuals in their 20s and 30s, when compared with older individuals in their 70s and above, exhibited higher risks of acute heart disease (adjusted HR, 1.70; 95% CI, 1.51–1.91; P < .0001 for age 20s, and adjusted HR, 1.52; 95% CI, 1.36–1.70; P < .0001 for age 30s). Additionally, people with high CCI scores, compared to those with a CCI of 0, displayed higher risks of acute heart disease (adjusted HR, 1.13; 95% CI, 1.04–1.24; P = 0.0063 for CCI = 1, and adjusted HR, 1.44; 95% CI, 1.22–1.71; P < .0001 for CCI ≥ 2). The Cox proportional hazards regression model revealed that there was no significant interaction effect between vaccine type and COVID-19 status (P for interaction = 0.1300).

**Table 2.** Risk of heart disease within 21 days after the first dose vaccination by vaccine type and COVID-19 with and without adjustment for confounding factors

	Univariable		Multivariable	
	Hazard ratio (95% CI)	P-value	Hazard ratio (95% CI)	P-value
*Vaccine Type*				
mRNA vaccine^a^	1.49 (1.39, 1.60)	<.0001	1.48 (1.35, 1.62)	<.0001
Others^b^	Ref		Ref	
*COVID–19^c^*				
Yes	4.08 (1.31, 12.64)	0.0150	3.56 (1.15, 11.04)	0.0280
No	Ref		Ref	
*Sex*				
Male	0.96 (0.89, 1.02)	0.1681	0.97 (0.91, 1.04)	0.3623
Female	Ref		Ref	
*Age group*				
10–19	1.11 (0.93, 1.33)	0.2621	1.03 (0.86, 1.24)	0.7321
20–29	1.80 (1.61, 2.02)	<.0001	1.70 (1.51, 1.91)	<.0001
30–39	1.50 (1.35, 1.68)	<.0001	1.52 (1.36, 1.70)	<.0001
40–49	1.08 (0.96, 1.21)	0.1846	1.06 (0.95, 1.19)	0.3095
50–59	0.93 (0.84, 1.03)	0.1746	0.89 (0.80, 0.99)	0.0376
60–69	0.90 (0.81, 0.99)	0.0400	1.15 (1.02, 1.30)	0.0210
70+	Ref		Ref	
*Charlson comorbidity index*				
2+	1.26 (1.06, 1.48)	0.0071	1.44 (1.22, 1.71)	<.0001
1	1.02 (0.94, 1.11)	0.6380	1.13 (1.04, 1.24)	0.0063
0	Ref		Ref	

a The mRNA vaccines consist of BNT162b2 and mRNA-1273.

b Other vaccines include AZD1222 (AstraZeneca), JNJ-78436735 (Janssen), and NVX-CoV2373 (Novavax).

c Time-varying covariate.

We further examined possible interactions among covariates and found a significant interaction effect between vaccine type and age group on the risk of acute heart disease (P < .0001). Table 3 shows the HRs for age groups within each vaccine type. The younger the age of mRNA vaccine recipients, the higher the risk of acute heart disease. On the other hand, among those who received other vaccines, there was a tendency for the risk of acute heart disease to increase with age. Additionally, among this group, individuals aged 30s and 40s exhibited significantly protective HRs (adjusted HR for age 30s, 0.62; 95% CI, 0.47–0.83; P = 0.0012 and adjusted HR for age 40s, 0.72; 95% CI, 0.56–0.94; P = 0.0139). Particularly, among mRNA vaccine recipients, compared to individuals aged 70 and above, those in their 20s showed the highest HR (adjusted HR for age 20s, 1.94; 95% CI, 1.71–2.21; P < .0001), followed by those in their 30s (adjusted HR for age 30s, 1.83; 95% CI, 1.60–2.08; P < .0001). Additionally, elevated risk was also observed among individuals in their 40s and 60s (adjusted HR for 40s, 1.25; 95% CI, 1.09–1.42; P = 0.0009 and adjusted HR for 60s, 1.36; 95% CI, 1.08–1.73; P = 0.0093, respectively). However, this increased risk was not observed among those in their 50s. On the other hand, among recipients of other vaccine types, a significant decrease in risk was observed for individuals in their 40s and 50s when compared to those aged 70 and above (adjusted HR for 40s, 0.62; 95% CI, 0.47–0.83; P = 0.0012 and adjusted HR for 50s, 0.72; 95% CI, 0.56–0.94; P = 0.0139, respectively).

**Table 3.** Risk of heart disease within 21 days after the first dose vaccination by age group and vaccine type *^d^* with adjustment for other covariates

	Hazard ratio (95% CI)	P-value
*Vaccine Type = mRNA vaccine^a^*		
*Age group*		
10–19	1.16 (0.96, 1.40)	0.1203
20–29	1.94 (1.71, 2.21)	<.0001
30–39	1.83 (1.60, 2.08)	<.0001
40–49	1.25 (1.09, 1.42)	0.0009
50–59	1.00 (0.88, 1.13)	0.9663
60–69	1.36 (1.08, 1.73)	0.0093
70+	Ref	
*Vaccine Type = Others^b^*		
*Age group*		
10–19	0.01 (0.00, >9,999)	0.9316
20–29	0.46 (0.19, 1.12)	0.0862
30–39	0.84 (0.65, 1.09)	0.1879
40–49	0.62 (0.47, 0.83)	0.0012
50–59	0.72 (0.56, 0.94)	0.0139
60–69	0.89 (0.77, 1.03)	0.1168
70+	Ref	
*COVID–19^c^*		
Yes	3.05 (1.13, 10.872)	0.0299
No	Ref	
*Sex*		
Male	0.98 (0.91, 1.04)	0.4719
Female	Ref	
*Charlson comorbidity index*		
2+	1.45 (1.23, 1.72)	<.0001
1	1.13 (1.04, 1.24)	0.0059
0	Ref	

a The mRNA vaccines consist of BNT162b2 and mRNA-1273.

b Other vaccines include AZD1222 (AstraZeneca), JNJ-78436735 (Janssen), and NVX-CoV2373 (Novavax).

c Time-varying covariate.

d Because a significant interaction effect between age group and vaccine type was observed (P < .0001), HRs for age group in each vaccine type are presented.

## Discussion

In this study, we examined the effect of COVID-19 status and vaccine type following the first dose on acute heart disease in the Korean population, using data from the NHIS COVID-19 database. We assessed the interaction effect between vaccine type and the COVID-19 status, as well as the interaction between age group and vaccine type. The study results showed that the risk of acute heart disease was higher among individuals who received mRNA vaccines compared to those who received other types of vaccines. Additionally, the risk was elevated among individuals who had COVID-19 within 21 days after the first vaccination compared to those who did not have a recorded COVID-19 during this period. There was no statistically significant interaction effect between vaccine type and COVID-19 status on the risk of acute heart disease. Due to the small number of individuals who developed acute heart disease among other vaccine recipients and had COVID-19 (only one case), the estimate of the HR for acute heart disease might be unstable. However, we observed that individuals vaccinated with mRNA vaccines had a higher risk of acute heart diseases compared to those who received other vaccines, and those with COVID-19 within 21 days after the first vaccination had a higher risk than those without. Furthermore, the interaction effect between the type of vaccine and age group revealed that among individuals who received mRNA vaccines, younger age was associated with a higher risk of acute heart disease.

These findings are consistent with previous studies, which have reported that cardiac AEs such as myocarditis or pericarditis shortly after mRNA vaccination are rare but possible, and they tend to occur more frequently in young patients [7, 17–20]. Complications, particularly myocarditis, have been reported following COVID-19 vaccination. In one study [18], the safety of BNT162b2 was evaluated in over 800,000 vaccinated participants with no prior history of confirmed vaccine-related AEs. The study reported a risk ratio (RR) of 3.2 (95% CI, 1.5–12.4) for developing myocarditis after vaccination, followed by pericarditis (RR, 5.4; 95% CI, 2.2–23.6), and myocardial infarction (RR, 4.5; 95% CI, 2.5–10.0) [18]. Furthermore, an examination of data from 2.5 million healthcare workers who had been vaccinated revealed an incidence rate of 2.13 cases of myocarditis per 100,000 individuals who had been administered at least one dose of the BNT162b2 vaccine [19]. As indicated by a recent study [21], even a mild instance of COVID-19 infection can escalate an individual’s susceptibility to cardiovascular complications for a minimum of 12 months following diagnosis. Researchers discovered that incidence rates of heart stroke and failure were considerably greater among individuals who had recovered from COVID-19 in comparison to individuals who had not contracted the disease [22].

Myocarditis emerges as a result of damage to the heart muscle, detectable through histological analysis or clinical assessment using a combination of clinical observations and diagnostic criteria [23, 24]. Most frequently, it ensues from viral infections, with enterovirus and adenovirus being conventionally associated with myocarditis [25]. Several clinical studies have indicated a correlation between COVID-19 and cardiovascular ailments, including myocardial injury, acute coronary syndrome, and arrhythmias [26–28]. Extensive research has been conducted on the genomic sequence and protein structure of the SARS-CoV-2 virus since its emergence [29, 30]. However, the precise process through which SARS-CoV-2 triggers harm to the heart muscle is not yet definitively understood. Several hypothesized explanations involve direct harm to heart muscle cells, widespread inflammation throughout the body, and immune responses mediated by interferons, among other factors [31]. Another suggested mechanism is molecular mimicry, in which external antigens in the SARS-CoV-2 virus exhibit sequence or structural resemblances to proteins in the heart, potentially leading to cross-reactive responses and inflammation [32–34]. Similarly, the precise mechanism underlying myocarditis subsequent to COVID-19 mRNA vaccination remains incompletely clarified [35].

Regarding the dataset we utilized, we believe that the cohort is representative of the vaccinated population, as the COVID-19 database includes randomly selected individuals among vaccine recipients. Concerning the completeness of COVID-19 status information in the dataset, the South Korean government recommended real-time PCR tests for COVID-19 diagnosis. Nasopharyngeal swabs are preferred to ensure the most accurate results; however, oropharyngeal swabs can be substituted if obtaining nasopharyngeal specimens proves challenging. Priority testing is available for individuals meeting the criteria for prioritized testing, and testing is provided free of charge at public health centres, screening clinics, and temporary testing sites. Additionally, testing is available at medical institutions, including screening clinics and general hospitals, where medical charges may apply.

The study has a limitation related to the use of an operational definition. In our analysis, an adverse event related to the vaccine was considered to be a new acute heart disease-related diagnosis occurring within 21 days of the first dose vaccination date. This operational definition was necessary because we lacked information on the specific association between COVID-19 vaccines and diagnostic codes in the NHIS COVID-19 database. Given that the recommended interval between the first and second vaccine doses for Pfizer/bioNTech was 21 days, we concentrated on infections occurring within 21 days after the initial vaccine dose. Such an approach can facilitate the analysis of the association with events transpiring shortly after vaccine administration, potentially leading to a more robust comprehension of the relationship between post-infection and the emergence of acute heart disease. However, it is widely recognized that the level of immunity and the quality of adverse effects recorded varies between the first and second doses in all vaccines. Thus, a cautious interpretation of the results is needed. Recent studies have indicated that the risk of myocarditis and pericarditis is highest after the second dose of mRNA vaccines. Therefore, investigating the interaction effects between COVID-19 and vaccine type specifically after the second dose would have been particularly useful. Nevertheless, the findings of this study offer valuable insights. Future research will address this gap by examining the interaction effects following the second and booster doses. When defining acute heart disease as a potential AE of vaccination, we excluded individuals diagnosed with acute heart disease before their vaccination date. This exclusion could be considered a partial limitation of the study. Some studies suggested that patients with heart failure who contract SARS-CoV-2 infection were at an increased risk of both cardiovascular and non-cardiovascular morbidity and mortality [36]. However, other studies have found no evidence of an increased risk of major adverse cardiovascular events following vaccination in patients with cardiovascular disease [37–39]. Nevertheless, our study revealed an increased risk of acute heart disease among individuals who were vaccinated with mRNA vaccines and did not have prior heart disease, as well as those with high CCI scores. In this study, the potential interference of other therapies (e.g., the use of angiotensin-converting enzyme (ACE) inhibitors) has not been analysed. It is challenging to precisely extract information about medication usage or concurrent therapies from the claim data. Nonetheless, examining the impact of other treatments or potential interactions with vaccine types or COVID-19 status would be interesting and important. Another limitation could be the restricted exploration of potential risk factors. We incorporated all accessible information related to the subjects from the database and accounted for pre-existing comorbidities using the CCI score. However, other potential risk factors such as BMI or occupational activity were not available for consideration.

However, our study has several strengths. First, the large sample size provided sufficient statistical power to investigate these rare outcomes that might not be feasible to assess in clinical trials. Secondly, the use of COVID-19 status as a time-dependent covariate improved the accuracy and granularity of estimates regarding the effect of this covariate on survival. Lastly, our study’s novelty lies in its assessment of the interaction between vaccine type and COVID-19 status on the incidence of acute heart disease, a topic that has been rarely explored in previous research.

We found no significant interaction effect between vaccine type and COVID-19 status on the risk of acute heart disease. Nevertheless, our observations indicated that individuals who received mRNA vaccines exhibited an increased risk of acute heart diseases compared to those who received other vaccines. Furthermore, individuals infected with COVID-19 faced a heightened risk compared to those who were not infected. Also, we observed a significant interaction effect between vaccine type and age group on the risk of acute heart disease. These findings may indicate the importance of considering alternative vaccine options for the younger population. However, it is conceivable that the observed association between vaccine type and age group could have been influenced by government vaccination policies and vaccine availability during different periods. Further research is essential to better understand the underlying mechanisms driving these interactions and to guide vaccination strategies effectively.

## Data Availability

The dataset used in the present study cannot be publicly shared in accordance with the national regulations for the protection of personal information. The Korea National Health Insurance Sharing Service (contact via https://nhiss.nhis.or.kr/) provides permission to access the NHIS COVID-19 database for qualified researchers.
